# Simultaneous Determination of 5 Flavonoids and 7 Saponins for Quality Control of Traditional Chinese Medicine Preparation Xinnaoshutong Capsule Using HPLC-VWD-ELSD

**DOI:** 10.1155/2017/3190185

**Published:** 2017-01-16

**Authors:** Jin Li, Yang Bai, Peng Zhang, Jun He, Jun Cao, Mingrui An, Li-min Hu, Xiu-mei Gao, Yan-xu Chang

**Affiliations:** ^1^Tianjin State Key Laboratory of Modern Chinese Medicine, Tianjin University of Traditional Chinese Medicine, Tianjin 300193, China; ^2^Tianjin Key Laboratory of Phytochemistry and Pharmaceutical Analysis, Tianjin University of Traditional Chinese Medicine, Tianjin 300193, China; ^3^College of Material Chemistry and Chemical Engineering, Hangzhou Normal University, Hangzhou 310036, China; ^4^Department of Surgery, University of Michigan, Ann Arbor, MI 48109, USA

## Abstract

Xinnaoshutong capsule (XC) is a traditional Chinese prescription derived from the ripe fruit of* Tribulus terrestris* L. (TT). Although XC has long been considered as an important herbal medicine, no analytical method of marker compounds for quality assessment is registered in the Chinese Pharmacopoeia. A simple analytical method of twelve marker components was developed and validated by HPLC-VWD-ELSD method. Chromatographic separation by HPLC was carried out on a Hedera ODS 2 column (4.6 × 250 mm, 5 *μ*m) by gradient elution with acetonitrile-water (0.1% formic acid) as the mobile phase. Various extraction conditions were optimized to achieve twelve marker compounds with faster extraction and higher recovery. The analytical condition was then validated in terms of the linearity, accuracy and precision, repeatability, and stability. The twelve markers were successfully quantified in 30 batches of commercial samples. The developed HPLC-VWD-ELSD could be used as a rapid and reliable way in the assessment and quality control of XC and TT.

## 1. Introduction

Xinnaoshutong capsule (XC), a traditional Chinese herbal formula made from the aqueous extract of air-dried ripe fruit of* Tribulus terrestris* L. (TT), has been traditionally used to treat vascular diseases such as cerebral arteriosclerosis [1]. It has been proven that it possesses the pharmacological effect of activating blood circulation to dissipate blood stasis, support healthy energy, and prevent cerebral arteriosclerosis [2]. The major chemical constituents of XC included saponins (furostanol saponins) and flavonoids. Saponin possessed diverse pharmacological activities such as antifungal [3], antihyperlipidemic [4, 5], antihypertensive [6], antidepressive [7], anticerebral ischemia [8], and anti-UVB-induced damage effects [9]. It also increases hormonal levels [10, 11], improves erectile function [12], and repels the dengue fever mosquito [13] and it is cytotoxic against most cell lines from leukemia [14]. Meanwhile, flavonoids have also demonstrated tremendous anticancer, antioxidant, anti-inflammatory, antibacterial, and antifungal effects [15–18]. Thus, the clinical and pharmacological effect of XC may be exerted through the combination and interaction of the above two types of constituents.

In order to guarantee XC to have an optimal therapeutic effect, the consistency of quality considered a crucial factor. This can be accomplished by maintaining a constant content of the bioactive compounds in XC. There are numerous chemical compounds in XC and their complexity and diversity make it difficult to determine the contents of all compounds contained in it. In addition, the composition of constituent compounds can be easily affected by the manufacturing process. Therefore, it is necessary to consider multiple marker constituents in XC to evaluate its quality.

Several approaches to the quantitative analysis of constituent components have been reported for the quality assessment of TT. Most studies of the chemical quantification of TT are focused on the simultaneous determination of fewer than five compounds (i.e., diosgen [19], terrestroneoside [20], saponin D [21], protodioscin [22] quercetin, kaempferol, and isorhamnetin [23] and quercetin, kaempferol, isorhamnetin, hecogenin, and tigogenin [24]). However, there is no officially established quality control method for XC till now. Hence, variations in the amounts of constituent compounds in XC preparations from different batches cannot be investigated.

**Figure 1:**
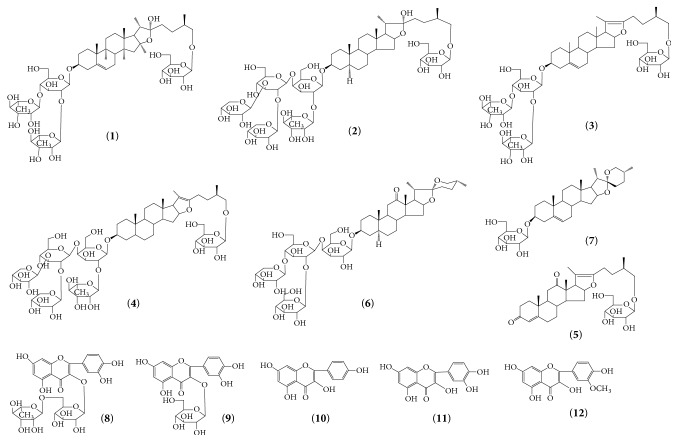
Chemical structures of the twelve bioactive components. (1) Protodioscin, (2) (25R)-26-O-*β*-D-glucopyranosyl-5*α*-furostan-3*β*,22*α*,26-triol-3-O-*β*-D-xylopyranosyl-(1→2)-[*β*-D-xylopyranosyl-(1→3)]-*β*-D-glucopyranosyl-(1→4)-[*α*-L-rhamnopyranosyl-(1→2)]-*β*-D-galactopyranoside, (3) Pseudoprotodioscin, (4) 26-O-*β*-D-glucopyranosyl-(25R)-5*α*-furostan-20(22)-en-3*β*,26-diol-3-O-{*β*-D-xylopyranosyl-(1→2)-[*β*-D-xylopyranosyl-(1→3)]-*β*-D-glucopyranosyl-(1→4)-[*α*-L-rhamnopyranosyl-(1→2)]-*β*-D-galactopyranoside}, (5) Terrestrinin A, (6) hecogenin-3-O-*β*-D-glucopyranosyl(1→2)-[*β*-D-xylopyranosyl-(1→3)]-*β*-D-glucopyranosyl-(1→4)-*β*-D-galactopyranoside, (7) Tibllin, (8) Rutin, (9) Isoquercitrin, (10) kaempferol, (11) quercetin, and (12) isorhamnetin.

In recent years, high performance liquid chromatography-diode array detector-evaporative light scattering detector (HPLC-DAD-ELSD) method was widely used in detecting the low ultraviolet absorption compounds, which is a simple, rapid, and accurate method and can reflect variations in the content of constituent components in herbal preparations and has been widely used for quality assessment of traditional Chinese herbal medicines and their preparations [25–27]. For the purposes of the quality of XC, an HPLC-VWD-ELSD (high performance liquid chromatography-variable wavelength detector-evaporative light scattering detector) was developed and validated to quantify some components. Rutin (8), Isoquercitrin (9), kaempferol (10), quercetin (11), and isorhamnetin (12) are typical flavonoids [23, 24] while protodioscin (1) and Pseudoprotodioscin (3) are typical saponins of* T. terrestris *[28, 29]. Till now, some publications on the isolation and structure elucidation of five saponins ((25R)-26-O-*β*-D-glucopyranosyl-5*α*-furostan-3*β*,22*α*,26-triol-3-O-*β*-D-xylopyranosyl-(1→2)-[*β*-D-xylopyranosyl-(1→3)]-*β*-D-glucopyranosyl-(1→4)-[*α*-L-rhamnopyranosyl-(1→2)]-*β*-D-galactopyranoside(2),26-O-*β*-D-glucopyranosyl-(25R)-5*α*-furostan-20(22)-en-3*β*,26-diol-3-O-{*β*-D-xylopyranosyl-(1→2)-[*β*-D-xylopyranosyl-(1→3)]-*β*-D-glucopyranosyl-(1→4)-[*α*-L-rhamnopyranosyl-(1→2)]-*β*-D-galactopyranoside}(4), Terrestrinin A (5) and hecogenin-3-O-*β*-D-glucopyranosyl(1→2)-[*β*-D-xylopyranosyl-(1→3)]-*β*-D-glucopyranosyl-(1→4)-*β*-D-galactopyranoside(6) do not offer any information about contents of* T. terrestris* and its related preparations XC [3, 29, 30]. This makes the proper selection of the marker components impossible for quality control of XC. The aphrodisiac properties of 1 [31] and glycolate oxidase inhibitory activity of 10 and 11 are reported in the literature [32]. In order to clarify distribution of chemical components in XC, these seven saponins and five flavonoids (Figure 1) were detected and selected as marker components for evaluating the consistency of quality of XC and TT.

In the present study, these twelve components were also simultaneously determined by developed and validated high performance liquid chromatography-variable wavelength detector-evaporative light scattering detector (HPLC-VWD-ELSD). It was demonstrated that a feasible HPLC-VWD-ELSD method is more reliable to determine sample contents as a means of developing quality control of TCM preparation.

## 2. Experimental

### 2.1. Reagents and Materials

XC (30 batches) were obtained from Jilin Aodong Pharmaceutical (Jilin, China). Reference compounds of (**2**) (25R)-26-O-*β*-D-glucopyranosyl-5*α*-furostan-3*β*,22*α*,26-triol-3-O-*β*-D-xylopyranosyl-(1→2)-[*β*-D-xylopyranosyl-(1→3)]-*β*-D-glucopyranosyl-(1→4)-[*α*-L-rhamnopyranosyl-(1→2)]-*β*-D-galactopyranoside, (4) 26-O-*β*-D-glucopyranosyl-(25R)-5*α*-furostan-20(22)-en-3*β*,26-diol-3-O-{*β*-D-xylopyranosyl-(1→2)-[*β*-D-xylopyranosyl-(1→3)]-*β*-D-glucopyranosyl-(1→4)-[*α*-L-rhamnopyranosyl-(1→2)]-*β*-D-galactopyranoside}, (5) Terrestrinin A, and (**6**) hecogenin-3-O-*β*-D-glucopyranosyl(1→2)-[*β*-D-xylopyranosyl-(1→3)]-*β*-D-glucopyranosyl-(1→4)-*β*-D-galactopyranoside were isolated from* Tribulus terrestris* L. in our laboratory. Reference compounds of (**1**) Protodioscin, (**3**) Pseudoprotodioscin, and (**7**) Tibllin were purchased from Zhongxin Innova (Tianjin, China), and (**8**) Rutin, (**9**) Isoquercitrin, (**10**) kaempferol, (**11**) quercetin, and (**12**) Isorhamnetin were purchased from Chengdu Musi Bio. Sci. and Tec. Co. Ltd. (Chengdu, China). The purity of each reference sample was determined to be above 98% by normalization of the peak area detected by HPLC-VWD-ELSD. Deionized water used for sample preparations and mobile phase was provided by a Milli-Q Academic ultrapure water system (Millipore, Bedford, MA, USA); acetonitrile and methanol were of HPLC grade (Tianjin Concord Science Co. Ltd., Tianjin, China). All other chemicals were of reagent grade.

### 2.2. Sample Preparation

#### 2.2.1. Reference Standard Solutions

All standard compounds were individually dissolved with methanol (approximately 1.0 mg mL^−1^). The mixed stock solution of twelve reference compounds was prepared by dissolving accurately weighted portions of the standards in methanol at a stock concentration of 0.3 mg mL^−1^ for compounds** 1**,** 3,** and** 6**, 0.6 mg mL^−1^ for compound** 2**, 2.1 mg mL^−1^ for compound** 4**, 0.78 mg mL^−1^ for compound** 5**, 0.24 mg mL^−1^ for compound** 7,** and 0.015 mg mL^−1^ or all other compounds. The mixed standard solution containing all the compounds was further diluted to obtain the working standard solution with a series of concentrations. All the standard solutions were stored at 4°C.

#### 2.2.2. Sample Solution Preparation

A weight of 0.7 g XC was accurately taken and then ultrasonically extracted for 40 min with 10 ml pure methanol, respectively. The sample solution was centrifuged at 14,000 rpm for 10 min and then an aliquot of the solutions was used for analysis, respectively.

#### 2.2.3. Instrument and Chromatographic Conditions

All experiments were performed on an Agilent 1200 HPLC system (Agilent Corp., USA) which consisted of a vacuum degasser, a binary pump, an auto sampler, a column compartment, and a VWD coupled with an ELSD (Alltech Associates, Deerfield, USA). Data acquisition and processing were operated by ChemStation software (Agilent Technologies, USA). The drift tube temperature for ELSD was 70°C, and the nebulizing gas flow rate was 1.8 L min^−1^. A constant flow rate of 1.0 mLmin^−1^ was employed throughout the analysis. All analysis was performed at 30°C. The chromatographic separations were achieved on a Hedera ODS 2 column (250 mm × 4.6 mm, 5 *μ*m). The mobile phases consisted of 0.05% formic acid (A) and acetonitrile (B) with a gradient elution as follows: 0–5 min, 5–15% (B); 5–10 min, 15–20% (B); 10–15 min, 20-20% (B); 15–20 min, 20–23% (B); 20–27 min, 23-23% (B); 27–30 min, 23–26% (B); 30–45 min, 26-26% (B); 45–50 min, 26–31% (B); 50–55 min, 31–35% (B); 55–65 min, 35-35% (B); 65–70 min, 35–39% (B); 70–75 min, 39-39% (B); 75–80 min, 39–50% (B); 80–85 min, 50–60% (B); 85–90 min, 60–90% (B); 90–95 min, 90–100% (B); 95–100 min, 100-100% (B). Then, a 10 *µ*L aliquot of the solution was injected into the HPLC-VWD-ELSD system for analysis. The detection wavelength was set at 360 nm.

### 2.3. Method Validation

A stock solution, which contained 12 analytes, was prepared with methanol and diluted to a series of appropriate concentrations for the construction of calibration curves. The solutions were brought to room temperature and an aliquot of 10 *μ*L was injected into HPLC for analysis. For the seven saponins, the calibration curves were calculated by linear regression of the double logarithmic plots of the peak area obtained by ELSD detection versus the concentration of the reference solution injected. For the five flavonoids, the calibration curves were calculated by linear regression of the peak area obtained by UV detection versus the concentration of the reference solution injected. The limits of detections (LODs) and quantifications (LOQs) under the present chromatographic conditions were determined by diluting the standard solution when the signal-to-noise ratios (S/N) of analytes were about 3 and 10, respectively. The S/N was calculated as the peak height divided by the background noise value. The analyses were performed six times on the same day. Recovery test was used to evaluate the accuracy of this method. The test was performed by adding accurately known amounts of the 12 standards into a certain amount (0.35 g) of XC separately. The spiked samples were then extracted, processed, and quantified in accordance with the methods mentioned above. Six replicates were performed for the test. The average recovery percentage was calculated by the formula: recovery (%) = (observed amount − original amount)/spiked amount × 100%. Accuracy presented as percent recovery, whereas the repeatability was calculated as the RSD of the signal. Interday accuracy and intermediate precision were determined as the average values of five intraday measurements taken over three days' period. The stability test was performed with one real sample solution which was stored at room temperature and analyzed at 0, 2, 4, 6, 12, and 24 hours.

## 3. Results and Discussion

### 3.1. Optimization of Extraction Procedure

In order to obtain an efficient extraction of flavonoids and saponins from XC, the variables of the extraction process were optimized. The different ultrasonical extraction time of 20 min, 40 min, and 60 min was investigated. The results suggested that the amounts of flavonoids and saponins are no significant difference with the extraction time of 40 min and 60 min while both of them were higher than extraction time of 20 min. Therefore, the optimum sample extraction condition was achieved by methanol for 40 min.

### 3.2. Optimization of HPLC-VWD Conditions

**Figure 2:**
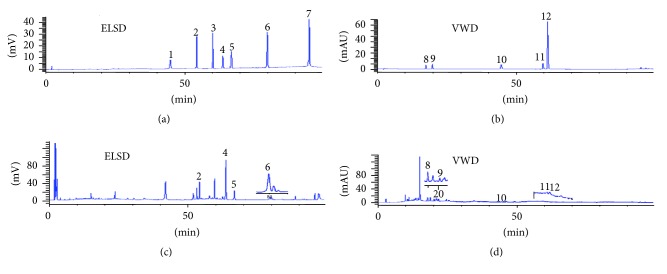
Typical HPLC-VWD-ELSD chromatograms for all of the reference compounds (a, b) and real sample (c, d). (1) Protodioscin, (2) (25R)-26-O-*β*-D-glucopyranosyl-5*α*-furostan-3*β*,22*α*,26-triol-3-O-*β*-D-xylopyranosyl-(1→2)-[*β*-D-xylopyranosyl-(1→3)]-*β*-D-glucopyranosyl-(1→4)-[*α*-L-rhamnopyranosyl-(1→2)]-*β*-D-galactopyranoside, (3) Pseudoprotodioscin, (4) 26-O-*β*-D-glucopyranosyl-(25R)-5*α*-furostan-20(22)-en-3*β*,26-diol-3-O-{*β*-D-xylopyranosyl-(1→2)-[*β*-D-xylopyranosyl-(1→3)]-*β*-D-glucopyranosyl-(1→4)-[*α*-L-rhamnopyranosyl-(1→2)]-*β*-D-galactopyranoside}, (5) Terrestrinin A, (6) hecogenin-3-O-*β*-D-glucopyranosyl(1→2)-[*β*-D-xylopyranosyl-(1→3)]-*β*-D-glucopyranosyl-(1→4)-*β*-D-galactopyranoside, (7) Tibllin, (8) Rutin, (9) Isoquercitrin, (10) kaempferol, (11) quercetin, and (12) isorhamnetin.

The optimization of HPLC conditions was carried out by using the mixed reference compound solutions. Considering the polarity differences of twelve compounds, different kinds of gradient elution of acetonitrile-water and gradient elution of acetonitrile-water (0%, 0.05%, and 0.1% formic acid), methanol-water (0%, 0.05%, and 0.1% formic acid) were investigated for XC, respectively. The results showed that it could achieve better separation on the flavonoid compounds with gradient elution of acetonitrile-0.05% formic acid aqueous solution. Then, nebulizing gas flow rate and evaporating temperature were optimized which were the two major instrument adjustments available for maximizing the detector response efficiency. Varying gas flow rates of 1.6, 1.8, and 2.0 Lmin^−1^ were investigated. The results revealed that the noise was decreased when the flow rate increased. However, the responses would be weakened following the increased flow rate. Thus, a moderate flow rate of 1.8 L min^−1^ was adopted to achieve the better performance. With respect to the drift tube temperature, a high baseline noise at low temperature, the optimal drift tube temperature, was determined at 70°C according to the data computed with the ELSD software. Typical HPLC-VWD-ELSD chromatograms for all of the reference compounds and real samples are shown in Figures 2(a) and 2(b).

### 3.3. Method Validation

The validation was performed following the requirements of the ICH Harmonized Tripartite Guideline. Some parameters including specificity, calibration range, linearity, sensitivity (LOD and LOQ), accuracy, repeatability, and intermediate precision were evaluated. The method was validated using real samples.

Linear regression of the twelve compounds showed good linear regression (all correlation coefficients >0.99) within the ranges of concentrations (Table 1). The LOD and the LOQ were less than 0.15 and 0.5 *μ*gmL^−1^ for flavonoids in VWD and were less than 4.3 and 13 *μ*gmL^−1^ for saponins in ELSD, respectively.

Intraday accuracy and repeatability were tested for twelve bioactive components. The solutions were prepared by adding known quantities of the analytes to the real samples. The analyses were performed six times on the same day. Accuracy presented as percent recovery, whereas the repeatability was calculated as the RSD of the signal. Interday accuracy and intermediate precision were determined as the average values of five intraday measurements taken over three days' period. Results are expressed in Table 2. Adequate recoveries (87.4–111%) and high precision (<4.60%) of the signal were found, thus demonstrating the reliability of the twelve bioactive components contents in XC determined by the present analytical method. For stability, no significant diminution of the peak area was detected for all analytes in 24 h period.

### 3.4. Sample Analysis

The developed HPLC-VWD-ELSD method was applied for simultaneous determination of twelve bioactive components in thirty batches of XC and two batches of TT and the results are presented in Table 3. The typical chromatograms of the samples detected by VWD and ELSD, respectively, are shown in Figures 2(c) and 2(d). As can be seen from Table 3, it was found that compounds 1 (Protodioscin), 3 (Pseudoprotodioscin), and 7 (Tibllin) were not detected in the XC and compounds 1 (Protodioscin), 3 (Pseudoprotodioscin), 7 (Tibllin), 10 (kaempferol), and 11 (quercetin) were not detected in TT. The content of compound** 4** (26-O-*β*-D-glucopyranosyl-(25R)-5*α*-furostan-20(22)-en-3*β*,26-diol-3-O-{*β*-D-xylopyranosyl-(1→2)-[*β*-D-xylopyranosyl-(1→3)]-*β*-D-glucopyranosyl-(1→4)-[*α*-L-rhamnopyranosyl-(1→2)]-*β*-D-galactopyranoside}) was significantly higher than other saponins in XC. Its content was (1.4%) higher than half of the total saponin (2.1%). The mean proportion of 26-O-*β*-D-glucopyranosyl-(25R)-5*α*-furostan-20(22)-en-3*β*,26-diol-3-O-{*β*-D-xylopyranosyl-(1→2)-[*β*-D-xylopyranosyl-(1→3)]-*β*-D-glucopyranosyl-(1→4)-[*α*-L-rhamnopyranosyl-(1→2)]-*β*-D-galactopyranoside that accounted for the total saponins was 65%. Similarly, content compound** 8** (Rutin) and compound** 9** (Isoquercitrin) were higher than other flavonoids in XC and TT. The mean proportion which contributed to the total flavonoids was 93.5%. For these consequences, the three compounds can be seen as the index components of quality control of XC and TT.

It has been reported that protodioscin (compound** 1**) was converted to 22-O-methylprotodioscin in methanol rapidly [28]. In our experiment, methanol was chosen as the solvent to dissolve the standard sample and XC samples, so peak 1 is 22-O-methylprotodioscin in the standard solution and sample solutions. There also has the same situation on (25R)-26-O-*β*-D-glucopyranosyl-5*α*-furostan-3*β*,22*α*,26-triol-3-O-*β*-D-xylopyranosyl-(1→2)-[*β*-D-xylopyranosyl-(1→3)]-*β*-D-glucopyranosyl-(1→4)-[*α*-L-rhamnopyranosyl-(1→2)]-*β*-D-galactopyranoside (compound** 2**), we also use compound** 2** to quantity methylated product. It was found that both 22-O-methylprotodioscin and protodioscin were not detected while compound 2 ranged from 0.1005% to 0.3683% in XC samples.

## 4. Conclusion

A reliable and simple method with HPLC-VWD-ELSD has been established and validated for simultaneous determination of twelve compounds in XC. The results of method validation illustrated that the presented method was precise, accurate, and sensitive as a practical technique for quantitative determination of multiple active components in XC. Twelve bioactive components including seven saponins and five flavonoids were selected as the chemical markers of XC to evaluate their quality of different batch samples. Compared with traditional HPLC-UV method, HPLC-VWD-ELSD could solve the problem of low UV absorption of saponins in order to achieve determination of multiple active components. Compared with the previous quality control methods of spectrophotometry, this method was more accurate, sensitive, and stable. Consequently, discrimination should be part of the quality control for TCMs. The proposed method could be readily utilized as a routine analysis and effective tool to evaluate the quality control of TCMs.

## Figures and Tables

**Table 1:** Linear regression data, LODs, and LOQs for the twelve compounds.

Compounds	Linear regression^a^	Linear range (*μ*g·mL^−1^)	*r* ^2^	LOD^b^ (*μ*g·mL^−1^)	LOQ^c^ (*μ*g·mL^−1^)
1	*y* = 1.6259*x* + 3.3728	50–300	0.9993	3.3	10
2	*y* = 1.5654*x* + 3.39	40–600	0.9981	1.3	4
3	*y* = 1.6565*x* + 3.5949	50–300	0.999	3.3	10
4	*y* = 1.554*x* + 3.0474	70–2100	0.995	2.3	7
5	*y* = 1.5726*x* + 3.2295	52–780	09956	4.3	13
6	*y* = 1.8064*x* + 3.6226	50–300	0.9985	3.3	10
7	*y* = 1.3249*x* + 3.6714	16–240	0.9983	0.64	2
8	*Y* = 17419*X* − 1.361	0.5–15	0.9962	0.13	0.5
9	*Y* = 19853*X* + 2.8905	0.5–15	0.9997	0.13	0.25
10	*Y* = 30126*X* + 3.3194	0.5–15	0.9993	0.13	0.5
11	*Y* = 43107*X* − 1.3252	0.15–15	0.9999	0.13	0.25
12	*Y* = 29297*X* + 0.2176	0.15–15	0.9999	0.15	0.25

^a^ *Y* is the peak area in UV chromatograms monitored at 360 nm, *X* is the compound concentration injected, and *y* and *x* are the logarithmic values of area and concentration injected in ELSD chromatograms.

^b^LOD refers to the limits of detection.

^c^LOQ refers to the limits of quantification.

**Table 2:** Intra- and interday accuracy, precision, and stability for the twelve compounds.

Components	Concentration (*μ*g·mL^−1^)	Intraday		Interday		Stability
		Accuracy^a^ (%)	Precision (RSD, %)	Accuracy (%)	Precision (RSD, %)	Precision (RSD, %)
1	0.1158	105	2.42	95.7	2.17	1.23
2	0.212	95.0	2.12	97.0	1.80	1.74
3	0.0947	110	1.67	95.9	2.66	2.52
4	1.6225	108	3.71	87.4	2.86	0.93
5	0.4594	113	1.39	97.1	1.37	1.81
6	0.2641	102	1.85	98.6	2.41	2.12
7	0.0324	111	4.54	100	2.90	2.67
8	0.0171	102	2.07	90.1	1.66	0.32
9	0.0060	110	2.58	88.3	2.54	0.59
10	0.001	104	4.77	110	1.31	1.18
11	0.0004	105	3.30	100	2.37	1.02
12	0.0019	109	3.64	105	1.04	1.61

^a^Recovery (%) = (amount determined − amount original)/amount spiked × 100%.

**Table 3:** Contents of nine components of samples from different batches of XC (%).

Batch	2	4	5	6	8	9	10	11	12	Saponin%	Flavone%
20110101139	0.2906	1.4035	0.4596	0.0151	0.0193	0.0072	0.0006	0.0004	0.0002	2.1687	0.0277
20110106081	0.2852	1.3240	0.4343	0.0185	0.0193	0.0070	0.0008	0.0004	0.0003	2.0620	0.0278
20100407101	0.3683	1.3842	0.5486	0.0222	0.0251	0.0105	0.0016	0.0006	0.0005	2.3234	0.0382
20110201029	0.2853	1.4192	0.4698	0.0181	0.0195	0.0074	0.0006	0.0003	0.0003	2.1923	0.0281
20110402021	0.2871	1.5198	0.4869	0.0178	0.0157	0.0056	0.0014	0.0005	0.0005	2.3115	0.0238
20100703001	0.1632	0.5924	0.1899	0.0212	0.0049	0.0016	0.0069	0.0021	0.0018	0.9667	0.0173
20100104118	0.3555	1.4975	0.5163	0.0208	0.0093	0.0033	0.0009	0.0005	0.0004	2.3901	0.0143
20101002104	0.2628	1.4878	0.3785	0.0168	0.0067	0.0024	0.0006	0.0003	0.0003	2.1459	0.0102
20110903074	0.2694	1.5167	0.4212	0.0187	0.0226	0.0081	0.0028	0.0009	0.0007	2.2260	0.0352
20101102056	0.2936	1.4332	0.3244	0.0189	0.0096	0.0039	0.0017	0.0006	0.0006	2.0702	0.0164
20110207058	0.3138	1.3034	0.3977	0.0198	0.0158	0.0064	0.0010	0.0005	0.0004	2.0347	0.0241
20100104118	0.3574	1.4958	0.5284	0.0207	0.0094	0.0035	0.0007	0.0004	0.0004	2.4023	0.0143
20110201022	0.3093	1.2911	0.4090	0.0199	0.0158	0.6430	0.0010	0.0005	0.0004	2.0293	0.6607
20110402020	0.3398	1.6046	0.5140	0.0176	0.0165	0.0062	0.0014	0.0005	0.0005	2.4759	0.0250
20090211016	0.1005	1.1903	0.3912	0.0215	0.0361	0.0133	0.0034	0.0010	0.0009	1.7035	0.0547
20110101137	0.2997	1.4651	0.5239	0.0170	0.0231	0.0092	0.0013	0.0004	0.0005	2.3057	0.0345
20100705001	0.1797	0.7505	0.2193	0.0178	0.0067	0.0023	0.0069	0.0015	0.0016	1.1673	0.0190
20100407100	0.3665	1.3997	0.5504	0.0204	0.0248	0.0103	0.0018	0.0006	0.0006	2.3369	0.0381
20100303124	0.3381	1.2121	0.4165	0.0198	0.0275	0.0104	0.0030	0.0009	0.0007	1.9865	0.0426
20110303061	0.3196	1.6128	0.4647	0.0165	0.0202	0.0072	0.0013	0.0005	0.0004	2.4136	0.0296
20110303046	0.3419	1.6801	0.4776	0.0181	0.0210	0.0073	0.0010	0.0005	0.0005	2.5176	0.0303
20101002075	0.2652	2.3379	0.3929	0.0153	0.0070	0.0026	0.0007	0.0003	0.0002	3.0113	0.0108
20110903058	0.2944	1.6131	0.4548	0.0189	0.0233	0.0085	0.0033	0.0009	0.0008	2.3811	0.0368
20090211015	0.3568	1.3093	0.4059	0.0189	0.0412	0.0140	0.0037	0.0013	0.0010	2.0909	0.0612
20100908090	0.2750	1.5758	0.4243	0.0147	0.0161	0.0059	0.0015	0.0006	0.0005	2.2898	0.0245
20100303124	0.3086	1.1683	0.3987	0.0161	0.0285	0.0101	0.0032	0.0009	0.0007	1.8917	0.0434
20100908042	0.2952	1.5704	0.4392	0.0161	0.0156	0.0061	0.0016	0.0006	0.0004	2.3208	0.0242
20110201056	0.2678	1.3067	0.4850	0.0142	0.0221	0.0087	0.0019	0.0004	0.0005	2.0737	0.0336
20110106068	0.2813	1.4765	0.3453	0.0149	0.0108	0.0044	0.0017	0.0005	0.0005	2.1180	0.0179
20101102072	0.2723	1.2733	0.3775	0.0133	0.0193	0.0069	0.0008	0.0003	0.0004	1.9363	0.0278
NM^a^	0.052	0.19	0.05	0.05	0.0023	0.00081	ND	ND	0.00018	0.342	0.00329
AH^a^	0.055	0.069	0.044	0.06	0.0025	0.0009	ND	ND	0.00047	0.228	0.00387

Note: compounds 1, 3, and 7 were not detected in the XC; compounds 1, 3, 7, 10, and 11 were not detected in TT; ND: not detected.

^a^NM: TT sample from neimenggu province, AH: TT sample from Anhui province.

## Abbreviations

HPLC-VWD-ELSD: High performance liquid chromatography-variable wavelength detector-evaporative light scattering detector
XC: Xinnaoshutong Capsule
RSD: Relative standard deviations
TCMs: Traditional Chinese medicines
LOD: Limit of detection
LLOQ: Lower limit of quantification.
